# A randomised clinical trial on cardiotocography plus fetal blood sampling versus cardiotocography plus ST-analysis of the fetal electrocardiogram (STAN^®^) for intrapartum monitoring

**DOI:** 10.1186/1471-2393-7-13

**Published:** 2007-07-26

**Authors:** Michelle EMH Westerhuis, Karel GM Moons, Erik van Beek, Saskia M Bijvoet, Addy P Drogtrop, Herman P van Geijn, Jan MM van Lith, Ben WJ Mol, Jan G Nijhuis, S Guid Oei, Martina M Porath, Robbert JP Rijnders, Nico WE Schuitemaker, Ingeborg van der Tweel, Gerard HA Visser, Christine Willekes, Anneke Kwee

**Affiliations:** 1 Department of Obstetrics and Gynaecology, University Medical Center Utrecht, The Netherlands; 2 Julius Center for Health Sciences and Primary Care, University Medical Center Utrecht, The Netherlands; 3 Department of Obstetrics and Gynaecology, Sint Antonius Hospital Nieuwegein, The Netherlands; 4 Department of Obstetrics and Gynaecology, VU Medical Center Amsterdam, The Netherlands; 5 Department of Obstetrics and Gynaecology, Tweesteden Hospital Tilburg, The Netherlands; 6 Department of Obstetrics and Gynaecology, Onze Lieve Vrouwen Gasthuis Amsterdam, The Netherlands; 7 Department of Obstetrics and Gynaecology, Academic Medical Center Amsterdam, The Netherlands; 8 Department of Obstetrics and Gynaecology, Maxima Medical Center Veldhoven, The Netherlands; 9 Department of Obstetrics and Gynaecology, Academic Medical Center Maastricht, The Netherlands; 10 Department of Obstetrics and Gynaecology, Jeroen Bosch Medical Center 's Hertogenbosch, The Netherlands; 11 Department of Obstetrics and Gynaecology, Diakonessenhuis Utrecht, The Netherlands; 12 Center for Biostatistics, Utrecht University, The Netherlands

## Abstract

**Background:**

Cardiotocography (CTG) is worldwide the method for fetal surveillance during labour. However, CTG alone shows many false positive test results and without fetal blood sampling (FBS), it results in an increase in operative deliveries without improvement of fetal outcome. FBS requires additional expertise, is invasive and has often to be repeated during labour. Two clinical trials have shown that a combination of CTG and ST-analysis of the fetal electrocardiogram (ECG) reduces the rates of metabolic acidosis and instrumental delivery. However, in both trials FBS was still performed in the ST-analysis arm, and it is therefore still unknown if the observed results were indeed due to the ST-analysis or to the use of FBS in combination with ST-analysis.

**Methods/Design:**

We aim to evaluate the effectiveness of non-invasive monitoring (CTG + ST-analysis) as compared to normal care (CTG + FBS), in a multicentre randomised clinical trial setting. Secondary aims are: 1) to judge whether ST-analysis of fetal electrocardiogram can significantly decrease frequency of performance of FBS or even replace it; 2) perform a cost analysis to establish the economic impact of the two treatment options.

Women in labour with a gestational age ≥ 36 weeks and an indication for CTG-monitoring can be included in the trial.

Eligible women will be randomised for fetal surveillance with CTG and, if necessary, FBS or CTG combined with ST-analysis of the fetal ECG.

The primary outcome of the study is the incidence of serious metabolic acidosis (defined as pH < 7.05 and Bd_ecf _> 12 mmol/L in the umbilical cord artery). Secondary outcome measures are: instrumental delivery, neonatal outcome (Apgar score, admission to a neonatal ward), incidence of performance of FBS in both arms and cost-effectiveness of both monitoring strategies across hospitals.

The analysis will follow the intention to treat principle. The incidence of metabolic acidosis will be compared across both groups. Assuming a reduction of metabolic acidosis from 3.5% to 2.1 %, using a two-sided test with an alpha of 0.05 and a power of 0.80, in favour of CTG plus ST-analysis, about 5100 women have to be randomised. Furthermore, the cost-effectiveness of CTG and ST-analysis as compared to CTG and FBS will be studied.

**Discussion:**

This study will provide data about the use of intrapartum ST-analysis with a strict protocol for performance of FBS to limit its incidence. We aim to clarify to what extent intrapartum ST-analysis can be used without the performance of FBS and in which cases FBS is still needed.

**Trial Registration Number:**

ISRCTN95732366

## Background

The aim of intrapartum fetal monitoring is to identify fetuses at risk for neonatal and long-term injury due to asphyxia. Although cardiotocography (CTG) is applied on a large scale, this technique is still subject to debate [1-3]. Long-term follow-up studies on intrapartum fetal heart rate monitoring have shown no or little benefit on neonatal outcome and a significant increase in operative deliveries [4-6]. Fetal blood sampling (FBS) can be used in addition to CTG, but requires expertise, is invasive, has to be repeated when CTG abnormalities persist and may cause complications [7,8]. As a consequence, it is not widely applied [9]. In The Netherlands, FBS is available in only 50 % to 70 % of the hospitals.

Because changes in the ST-segment of the electrocardiogram (ECG) are related to metabolic acidosis of the fetus, detection of changes in this part of the fetal ECG, in combination with CTG, is a non-invasive and promising alternative for FBS [10,11]. A recent study in more than 600 women showed that ST-changes were present in all cases with severe metabolic acidosis and that CTG plus ST-analysis was more specific in detecting fetal acidemia than CTG alone [12].

Two large randomised trials comparing CTG and ST-analysis of the fetal ECG showed a decrease in metabolic acidosis and interventions for fetal distress in favour of the CTG plus ECG-group [13,14]. The rate of infants with encephalopathy was also significantly lower in the CTG plus ECG-group [15]. However, in both trials FBS was still frequently performed in both arms. Hence, it remains difficult to conclude if the observed improved outcome was indeed due to management to address metabolic acidosis based on ST-analysis results or on FBS results.

Just before the initiation of the present study a third and much smaller randomised trial on ST-analysis versus conventional CTG appeared. This study showed, although not significant, an opposite effect on the incidence of neonatal acidemia or metabolic acidosis, compared to previous trials [16]. The caesarean section and vacuum outlet rate was comparable in both groups. The only significant difference was the incidence of FBS, which was much lower in the ST-analysis group (7.0%) than in the CTG group (15.6%). The results of this trial further stress the need for subsequent research.

In this paper we describe the study protocol of a recently started randomised trial to compare the cost-effectiveness between conventional CTG versus ST-analysis of the fetal ECG, in which the use of fetal blood sampling in the ST-analysis group was a priori restricted to well-defined situations.

### Rationale for ST-analysis (STAN^®^)

In adult cardiology, ST-analysis of ECG is performed to assess and diagnose myocardial insufficiency. The STAN^® ^concept is similarly based on the association between the ST-interval of fetal ECG and the function of the fetal myocardium during stress. The fetal heart and brain are equally sensitive to oxygen deficiency. As a result, the information relating to the function of the fetal myocardium provides an indirect measurement of the condition of the fetal brain during labour.

**Figure 1:**
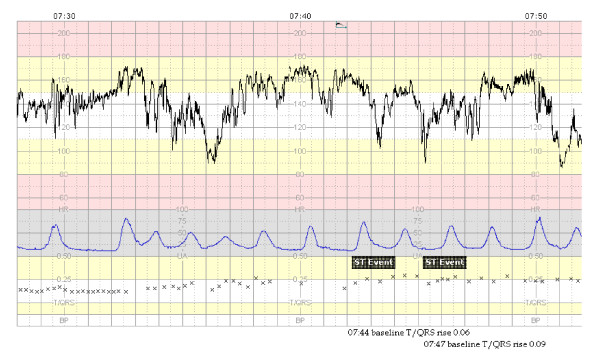
**Example of a STAN-registration with an abnormal CTG and two significant ST-events**. (1 cm/minute).

The changes in fetal ECG associated with fetal distress are either an increase in T-wave, quantified by the ratio T-wave to QRS-amplitude (T/QRS ratio), or a biphasic ST-segment (Figure 1). An increase in T-wave and subsequently in T/QRS-ratio has been associated with a catecholamine surge, activation of β-adrenoreceptors, myocardial glycogenolysis, and metabolic acidosis [10,11,17]. A biphasic shape of the ST-segment is related to two situations. First, it may occur when the fetal heart is exposed to acute hypoxic stress whereby it has had no time to respond to hypoxia or second, when the fetal heart has a reduced capacity to respond due to stress situations and lack of or already utilized resources. Biphasic ST-changes of the fetal ECG have been associated with disturbances in heart muscle function, infection or malformations [10,11,17].

The integrated CTG and fetal ECG monitor, i.e. the so-called STAN^®^-monitor, is a device that automatically analyses the fetal ECG through a scalp electrode applied to the fetal head [18]. STAN^® ^guidelines are based on an integrated CTG and fetal ECG interpretation. According to the STAN^® ^guidelines ST-changes are only thought to be of clinical relevance if they coincide with intermediate or abnormal CTG traces (Table 1). In case of a normal or (pre)terminal CTG, the high sensitivity of the CTG only, is considered sufficient to ignore abnormalities in the ECG.

**Table 1:** Classification of cardiotocographic patterns according to FIGO-Guidelines

**Cardiotocographic classification**	**Baseline heart frequency**	**Variability Reactivity**	**Decelerations**
Normal	110–150 beats/min	5–25 beats/min Accelerations	Early decelerations Uncomplicated variable decelerations with a duration of < 60 sec and a beat loss of < 60 beats/min
Intermediary*	100–110 beats/min 150–170 beats/min Short bradycardia episode	> 25 beats/min without accelerations < 5 beats/min for > 40 min	Uncomplicated variable decelerations with a duration of < 60 sec and a beat loss of > 60 beats/min
Abnormal	150–170 beats/min and reduced variability >170 beats/min	< 5 beats/min for > 60 min Sinusoidal pattern	Repeated late decelerations Complicated variable decelerations with a duration of > 60 sec
Preterminal	Total lack of variability and reactivity with or without decelerations or bradycardia		

* Combination of several intermediary observations will result in an abnormal CTG.

In spite of the above-mentioned evidence on the potential improvement in fetal surveillance using intrapartum ST-analysis [12-14], there is at present still a dilemma for gynaecologists on its true clinical value (i.e. without the use of FBS), let alone that its cost-effectiveness is known. ST-analysis requires financial investment, not only related to the procure of the ST-monitor (34000 Euro per monitor), but also related to the necessity of repeated training of labour ward personnel [19].

Our randomised trial aims to quantify this cost-effectiveness of fetal monitoring with CTG plus ST-analysis of the fetal ECG, as compared to conventional CTG plus FBS.

## Methods/Design

### Aims

The primary aim of our randomised trial is to quantify whether the incidence of metabolic acidosis is decreased by the use of a strategy of fetal monitoring with CTG plus ST-analysis of the fetal ECG, compared to usual care consisting of CTG plus FBS, when indicated.

Secondary aims are to quantify the cost-effectiveness of CTG in combination with non-invasive ST-analysis of the fetal ECG for fetal monitoring during labour as compared to usual care and the differences in incidence of operational deliveries and performance of FBS across both groups.

### Study design

The trial is a pragmatic randomised diagnostic study. We have choosen a randomised design because ST-analysis of the fetal ECG is an example of a new test that might provide better or other information, potentially leading to other treatment choices than the existing reference (CTG plus FBS). In such a situation there is an indication to do a randomised study rather than a conventional accuracy study in which ST-analysis is compared to CTG plus FBS, to quantify its value on patient outcome [20-22]. A design with randomisation at the moment that the CTG group and the ST-analysis group provide discordant test results was not feasible, as this would require randomisation at the moment of medical emergency [21].

### Setting

This study is set in the Dutch Obstetric Consortium, a collaboration of obstetric clinics in the Netherlands. The study will be carried out in nine hospitals, including academic hospitals and non-academic teaching hospitals [23].

### Participants/Eligibility criteria

Women will be eligible if they are in labour with a singleton fetus in vertex position, a gestational age ≥ 36 weeks and a medical indication for electronic fetal monitoring. A medical indication is defined by either a high-risk pregnancy, induction or augmentation of labour, epidural anaesthesia, meconium stained amniotic fluid or non-reassuring fetal heart rate.

#### Exclusion criteria

Exclusion criteria: breech presentation, twin pregnancies, maternal age < 18 years or absent informed consent.

### Procedures, recruitment, randomisation and collection of baseline data

Eligible women will receive the patient study information around 36 weeks of gestation, in the outpatient clinic. Women, having their prenatal controls under supervision of a midwife and being referred to the hospital during labour, will be informed and asked for consent by the attending doctor or midwife at arrival at the hospital.

After consent, women are randomised through a computer-generated randomisation sequence. Stratification will be applied for centre and parity (no previous vaginal delivery versus one or more previous vaginal deliveries). Randomisation will be 1:1 for either monitoring by CTG plus ST-analysis or CTG plus FBS. Both strategies will be performed according to strict protocols (see below).

Women who decide not to participate in the study will be asked for their reasons of refusal. They will be monitored with CTG and, if indicated, FBS.

In each centre an independent gynaecologist will be responsible for the centre specific data collection. Per centre a research nurse or midwife will monitor the protocol also via patient meetings and feedback on potential protocol violations.

At baseline, demographic, past obstetric and medical history data will be recorded for all women.

### Intervention

#### Control group: CTG

In women randomised to the control group, a scalp electrode will be applied to the fetal head and connected to the conventional CTG-monitor conform routine practice. CTG-interpretation will be guided by the FIGO guidelines (Table 1) [24]. Fetal blood sampling is recommended in case of an intermediate or abnormal CTG.

If the pH of the first FBS measurement is below 7.20 delivery is recommended, unless the cause of fetal distress can be alleviated. If the pH is between 7.20 and 7.25, FBS will be repeated after 30 minutes. If the pH is above 7.25, FBS is repeated according to the consecutive CTG pattern on discretion of the attending doctor or midwife.

#### Intervention group: CTG and ST-analysis

In women randomised to the intervention group, a scalp electrode will be applied to the fetal head and connected to the STAN^®^-monitor. This electrode will allow both standard fetal heart rate monitoring (CTG) as well as ST-analysis of the fetal ECG. The CTG will be classified as normal, intermediate, abnormal or preterminal according to the FIGO-guidelines for fetal heart rate monitoring (Table 1) [24]. Clinical management will be supported by computerised ST waveform assessment and will be guided by the STAN^®^-guidelines, indicating when intervention is recommended [25]. The ST log automatically alerts the attending doctor or midwife if a significant ST-event occurs. Delivery is recommended when there are significant ST-changes unless the cause of fetal distress can be alleviated (Table 2).

**Table 2:** STAN^® ^clinical guidelines: ST-changes that prompt clinical intervention, such as delivery or solving a cause of fetal distress

	**Intermediary CTG**	**Abnormal CTG**
Episodic T/QRS-rise (duration < 10 min)	Increase > 0.15 from baseline	Increase > 0.10 from baseline
Baseline T/QRS-rise (duration ≥ 10 min)	Increase > 0.10 from baseline	Increase > 0.05 from baseline
Biphasic ST (a component of the ST-segment below the baseline)	Continuous >5 min or >2 episodes of coupled Biphasic ST type 2 or 3	Continuous >2 min or >1 episode of coupled Biphasic ST type 2 or 3

The ST log requires 20 minutes recording for automatic ST analysis to start. A decrease in signal quality with insufficient number of T/QRS measurements requires manual data analysis.

Preferably, FBS will not be performed in this group. This, however, does not suffice in all cases. The aim is to keep the performance of FBS as low as possible, limited to three well-defined situations: 1) poor signal quality of the fetal ECG in combination with an abnormal CTG; 2) STAN^®^-registration starts with an abnormal CTG; 3) 60 to 90 minutes of abnormal CTG recording during first stage of labour without ST-events and the attending doctor decides not to intervene by caesarean section. In the second stage of labour it is advised to perform an instrumental delivery (if possible) in case of the before mentioned situations.

Furthermore, we will also consider the incidence of FBS in both groups as secondary outcome.

### Follow up

Children who were admitted to the neonatal intensive care or high care after delivery because of birth asphyxia or other delivery trauma, will be checked upon at 6 months post-delivery. These check-ups are part of the regular controls of children.

### Outcome measures

#### Primary outcome measures

The primary outcome will be the incidence of serious metabolic acidosis defined as a pH < 7.05 and a BD_ecf _> 12 mmol/l in the umbilical cord artery [26].

#### Secondary outcome measures

Secondary outcomes are:

1. Instrumental delivery rate because of fetal distress, failure to progress or a combination of the two

2. Neonatal outcome defined as Apgar scores < 4 after 1 minute and/or < 7 after 5 minutes

3. Need for admission to neonatal medium or intensive care unit

4. Incidence of performance of FBS in both groups

5. Cost-effectiveness of both monitoring strategies in general and across hospitals.

### Statistical issues

#### Sample size

The sample size calculation is based on the primary endpoint, which is metabolic acidosis in the umbilical cord artery.

Although in the two previous randomised trials the incidence of metabolic acidosis decreased from 1.5 % to 0.5 % in favour of the CTG + ST-analysis group [13,14], we assume that the incidence of metabolic acidosis in our higher-risk population (women delivering in the hospital with a medical indication/risk factor) is higher and estimated on 3.5 %, as found in our preliminary study [12]. Based upon the numbers of the largest clinical trial with a relative risk of 0.5 the required sample size would then yield 2400 cases (1200 per arm), using an alpha of 0.05 (2-sided) and a power of 0.80 [13]. However, soon after the start of our study a third randomised clinical trial appeared, although much smaller and non-significant, but yielding an opposite effect [16]. A meta-analysis of the three clinical trials showed the varying relative risks of 0.5, 0.4 and 2.4 [27]. Hence, to be conservative we used the meta-analysis overall relative risk of 0.6 for our power calculation, implying a reduction of metabolic acidosis, in favour of ST-analysis, from 3.5% to 2.1%. With an alpha of 0.05, a two-sided test – given conflicting results in the literature – and a power of 0.80, about 4638 women should be randomised (2319 per arm). Accounting for 10% loss to follow-up, the study requires inclusion of about 5100 women in order to obtain 4638 analysable cases.

#### Data analysis

The analysis of the primary endpoint will follow the intention to treat principle. Since this is a randomised trial, we would anticipate minimal differences in baseline characteristics. The relative risk with 95% confidence interval of metabolic acidosis in the CTG plus ST-analysis group compared to the CTG (plus FBS) group will be calculated, accounting for the stratified randomisation by centre and parity. Relative risks and 95% confidence intervals will also be calculated for the dichotomous secondary outcomes, i.e. instrumental delivery rate, neonatal outcome, need for neonatal admission and incidence of performance of FBS across both groups.

The following planned subgroup analyses will be performed: analysis according to risk pregnancies such as women with insulin dependent diabetes mellitus, fever during delivery and start of a STAN^®^-registration with an abnormal trace.

Missing data rarely occur at random. Simply excluding subjects with missing values thus not only lead to loss of statistical power but also to biased study results. To decrease bias and increase statistical efficiency we will therefore impute missing values rather than perform complete case analysis only. This will be done using single and multiple imputation methods [28-30].

### Economic evaluation

The economic evaluation is primarily a cost-effectiveness analysis (CEA) to find the optimal strategy as the one with the most favourable trade-off between avoided adverse neonatal outcome (fetal distress/metabolic acidosis) and difference in cost.

For this purpose, the process of care is distinguished into two cost stages (delivery/childbirth stage and postnatal stage) and three cost categories (direct medical costs [all costs in the health care sector, such as type of intervention and maternal and fetal monitoring, lab tests, costs associated with intrapartum complications, costs of training, maternal and neonatal care in the postnatal stage], direct non-medical costs [costs outside the health care sector that are affected by health status or health care], and indirect costs [productivity costs, costs of sick leave]).

Valuations of direct medical resources are estimated as cost per unit estimates comprising 'true' economic costs, i.e. including shares of fixed costs and hospital overheads. Cost per unit is estimated for at least one teaching and one non-teaching hospital. Direct medical volumes outside the hospital and direct non-medical volumes are valued using national reference prices [31]. Indirect costs are quantified according to the friction cost method. Study specific costs are excluded from analysis.

As we anticipate a reduction of metabolic acidosis with the use of CTG plus ST-analysis compared to CTG plus FBS, the economic analysis is planned to be a cost-effectiveness analysis. We will use bootstrap sampling to calculate 95% confidence intervals around the cost-effectiveness ratios. Sensitivity analysis will be used to explore the effect of variation of several key factors.

### Baseline obstetric characteristics of first 500 randomised patients

Baseline obstetric characteristics of the first 500 enrolled patients in the study are shown in table 3.

**Table 3:** Baseline obstetric characteristics of the first 500 randomised patients in clinical trial. All values are absolute numbers (%) or mean (SD).

	CTG group	CTG + ST group
	
	**n **= 254	**n **= 246
**Age at delivery **(yrs)	32 ± 5	32 ± 5
**Nulliparous**	120 (47.2%)	130 (52.8%)
**Previous ceasarean section**	45 (17.7%)	30 (12.2%)
**Gestational age at delivery (wks)**	40 ± 1.5	40 ± 1.5
**Prolonged pregnancy **(> 42 gestational wks)	31 (12.2%)	24 (9.8%)
**Induction of labour**	82 (32.3%)	87 (35.4%)
**Epidural analgesia**	79 (31.1%)	73 (29.7%)
**Meconium-stained amniotic fluid**	62 (24.4%)	61 (24.8%)
**Oxytocin augmentation**	146 (57.5%)	156 (63.4%)
**Birthweight (gr)**	3506 ± 528.7	3501 ± 508.1

### Ethical considerations and Safety Committee

The study protocol has been approved by the Medical Ethical Committee of the University Medical Center Utrecht, Utrecht, The Netherlands (05/157-K). Written informed consent will be obtained from each participating patient.

Serious Adverse Events (SAE) are defined as "metabolic acidosis (arterial pH < 7.00 and BD_ecf _>12 mmol/L) and admission to a Neonatal Intensive Care Unit (NICU)" or "an Apgar score < 7 after 5 minutes and admission to a NICU". Given the conflicting results in the literature, all SAE's will be reported to the Data Safety Monitoring Committee, consisting of an independent gynaecologist, neonatologist, epidemiologist and a biostatistician. This committee will consider the reported incidence of SAE's at regular intervals to determine whether there are significantly more serious adverse events in the STAN-group and, if so, whether the study should be discontinued. For this purpose the computer program PEST, version 4, will be used [32].

### Concluding remarks

This study is the first randomised trial to quantify the cost-effectiveness of ST-analysis of the fetal ECG during labour and the first to achieve data about the use of ST-analysis with fetal blood sampling performed only in well-defined circumstances. This study aims to provide additional evidence on the true clinical value of intrapartum ST-analysis and also the clinical indications in which fetal blood sampling still might be necessary in addition to ST-analysis.

## Abbreviations

CTG – cardiotocogram

ECG – electrocardiogram

FBS – fetal blood sampling

STAN – ST ANalysis

NICU – neonatal intensive care unit

## Competing interests

The author(s) declare that they have no competing interests.

## Authors' contributions

AK, BM, GV, IT, KM, and MW were involved in conception and design of the study. AK, KM and MW drafted the manuscript.

All authors have read and given final approval of the final manuscript.

## Pre-publication history

The pre-publication history for this paper can be accessed here:


